# Supplementary material for: The thermal response of soil microbial methanogenesis decreases in magnitude with changing temperature
Source: Nat Commun. 2020 Nov 12;11:5733. doi: 10.1038/s41467-020-19549-4 (PMC7665204; doi:10.1038/s41467-020-19549-4)
Supplement: Supplementary file 1 — Supplementary Information [file 41467_2020_19549_MOESM1_ESM.pdf]

Supplementary Information

The thermal response of soil microbial methanogenesis decreases in magnitude with changing temperature

Chen et al. 2020

Supplementary Figures

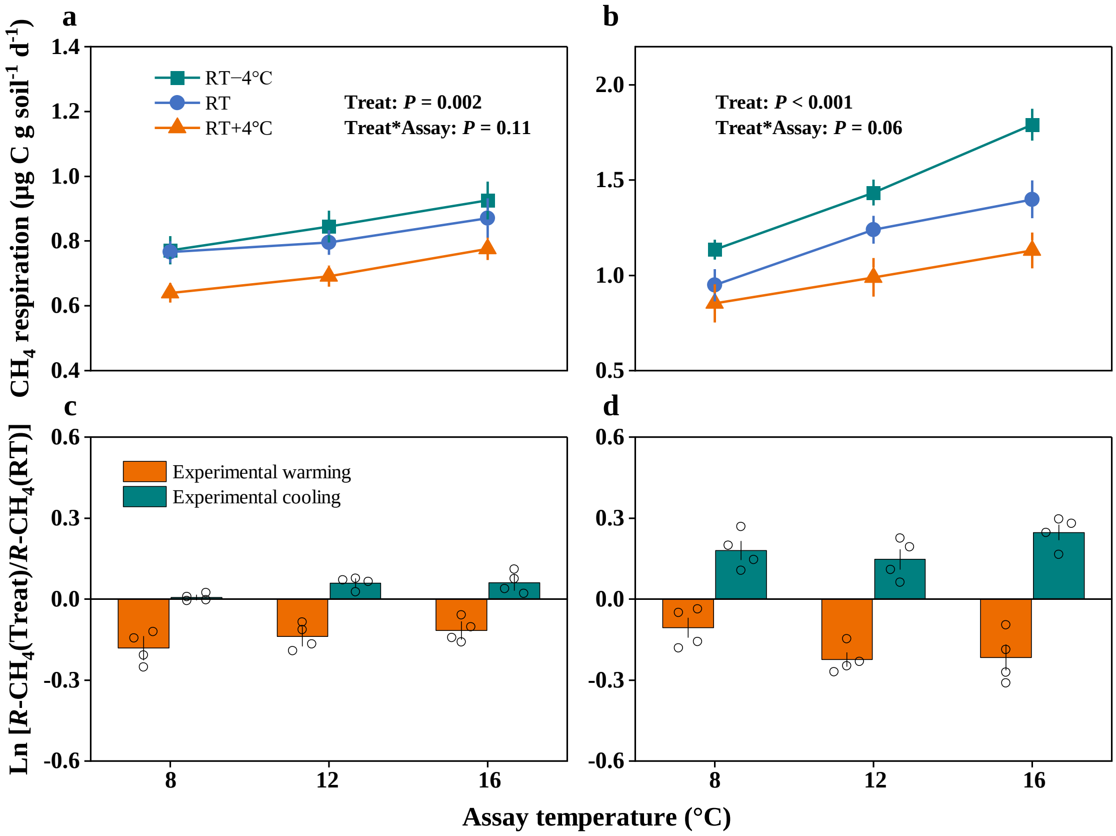

Supplementary Figure 1. The soil CH_4 respiration rate decreased under warming and increased under cooling. **a**, **c** The Greater Khingan Range; **b**, **d** The Tibetan Plateau. A linear mixed-effects model was used to test for incubation and assay temperature effects on the soil CH_4 respiration, the fixed factors were the incubation temperature (RT−4°C, RT, and RT+4°C; Treat) and assay temperature (8, 12, and 16°C; Assay), and the random factor was the spatial replicates within each wetland type in **a** and **b**. For clarity the values in **c** and **d** are the natural log of the treatment (RT−4°C or RT+4°C): control (RT) ratios of soil CH_4 respiration (*R*-CH_4) following 160 days of experimental warming and cooling. Ratios > 0 indicate a greater *R*-CH_4 rate in the treatment than in the control, and ratios < 0 indicate the reverse. RT, reference temperature (12°C). Data are presented as mean values ± SEM, n = 4 independent soil samples.

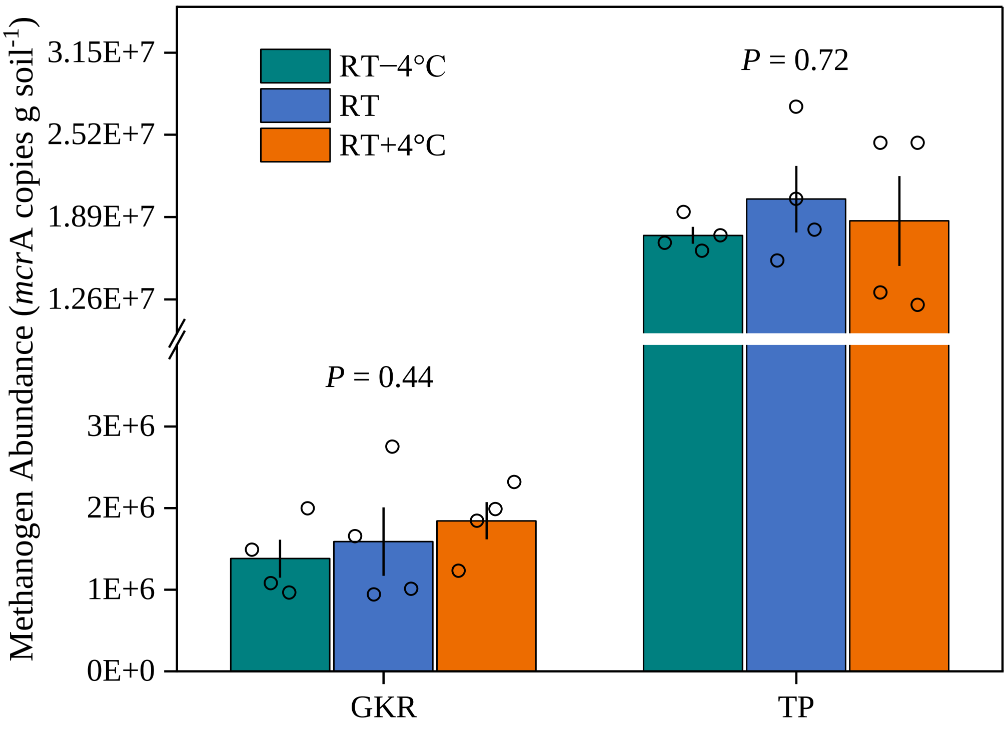

Supplementary Figure 2. Methanogen abundance of wetland soils under different thermal incubations. The abundance of *mcr*A gene copies was used as a proxy for biomass of methanogens. RT, reference temperature (12°C). GKR, the Greater Khingan Range; TP, the Tibetan Plateau. Ordinary one-way ANOVA for comparing the three thermal treatments. Data are presented as mean values ± SEM, n = 4 independent soil samples.

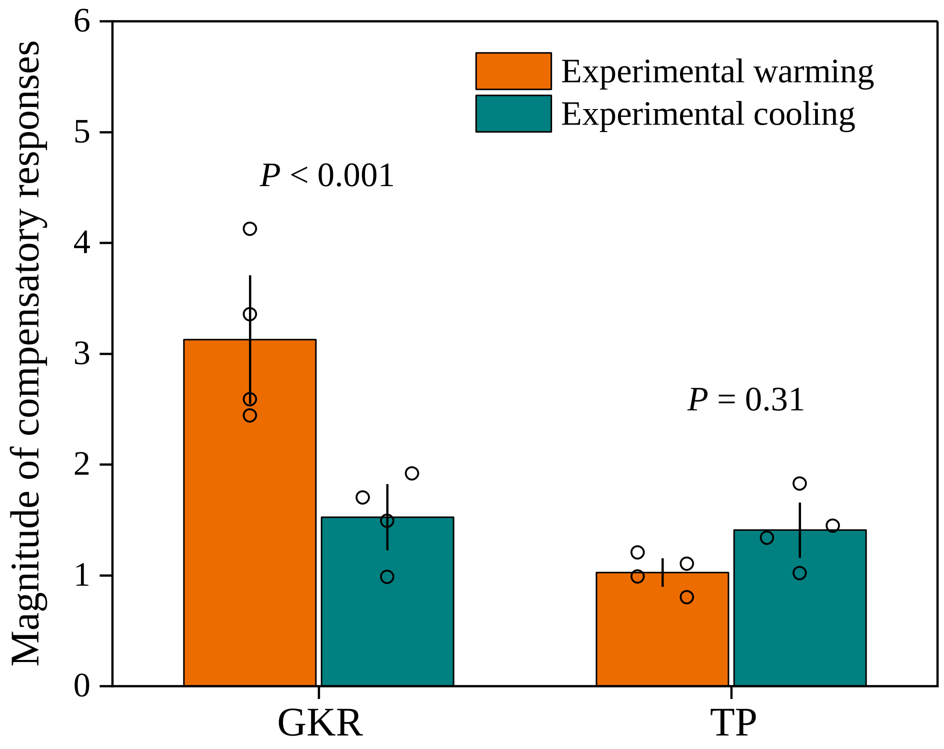

Supplementary Figure 3. Magnitude of compensatory responses exhibited by soil mass-specific CH_4 respiration in response to experimental warming or cooling by 4°C. For details on the calculation of the magnitude of compensatory responses, see Supplementary Fig. 8. GKR, the Great Khingan Range; TP, the Tibetan Plateau. Two-tailed paired *t*-tests. Data are presented as mean values ± SEM, n = 4 independent soil samples.

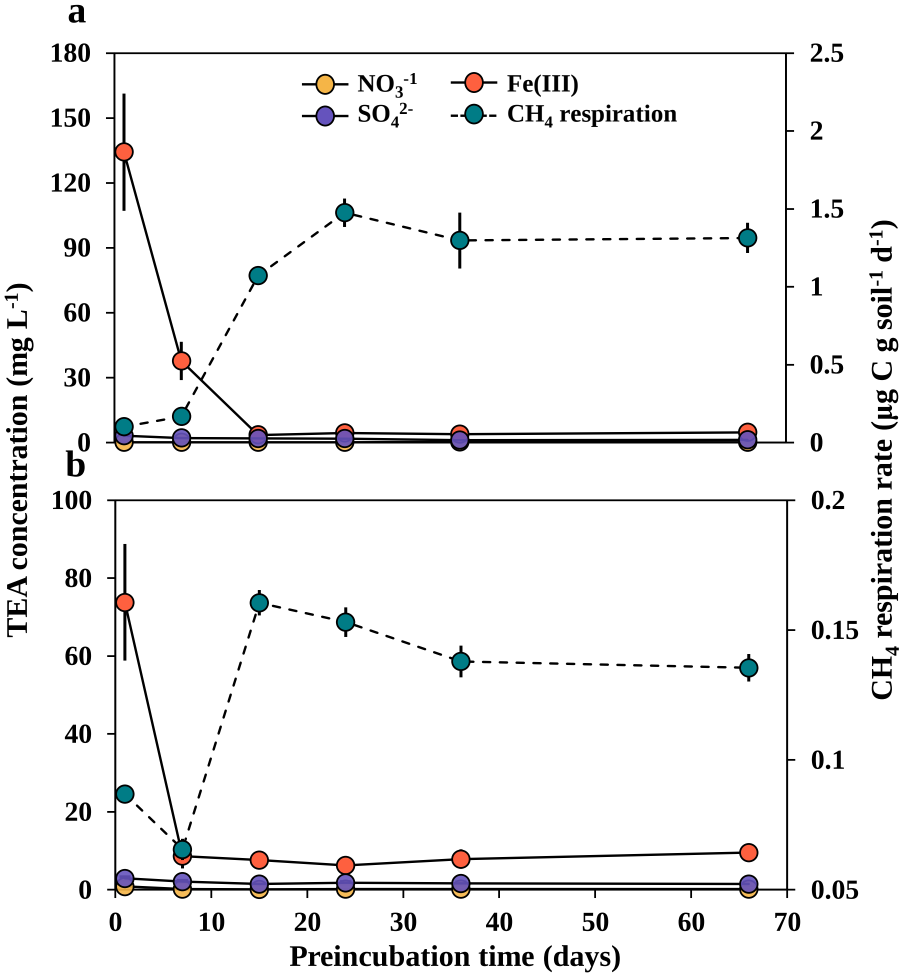

Supplementary Figure 4. Changes in the concentrations of inorganic terminal electron acceptors and soil CH_4 respiration rates during the preincubation period. The CH_4 respiration rate gradually stabilized with the depletion of inorganic terminal electron acceptors (TEAs). **a**, the Great Khingan Range; **b**, the Tibetan Plateau. The main TEAs were nitrate (NO_3^-), ferric iron [Fe(III)], and sulfate (SO_4^{2-}). Data are presented as mean values ± SEM, n = 4 independent soil samples.

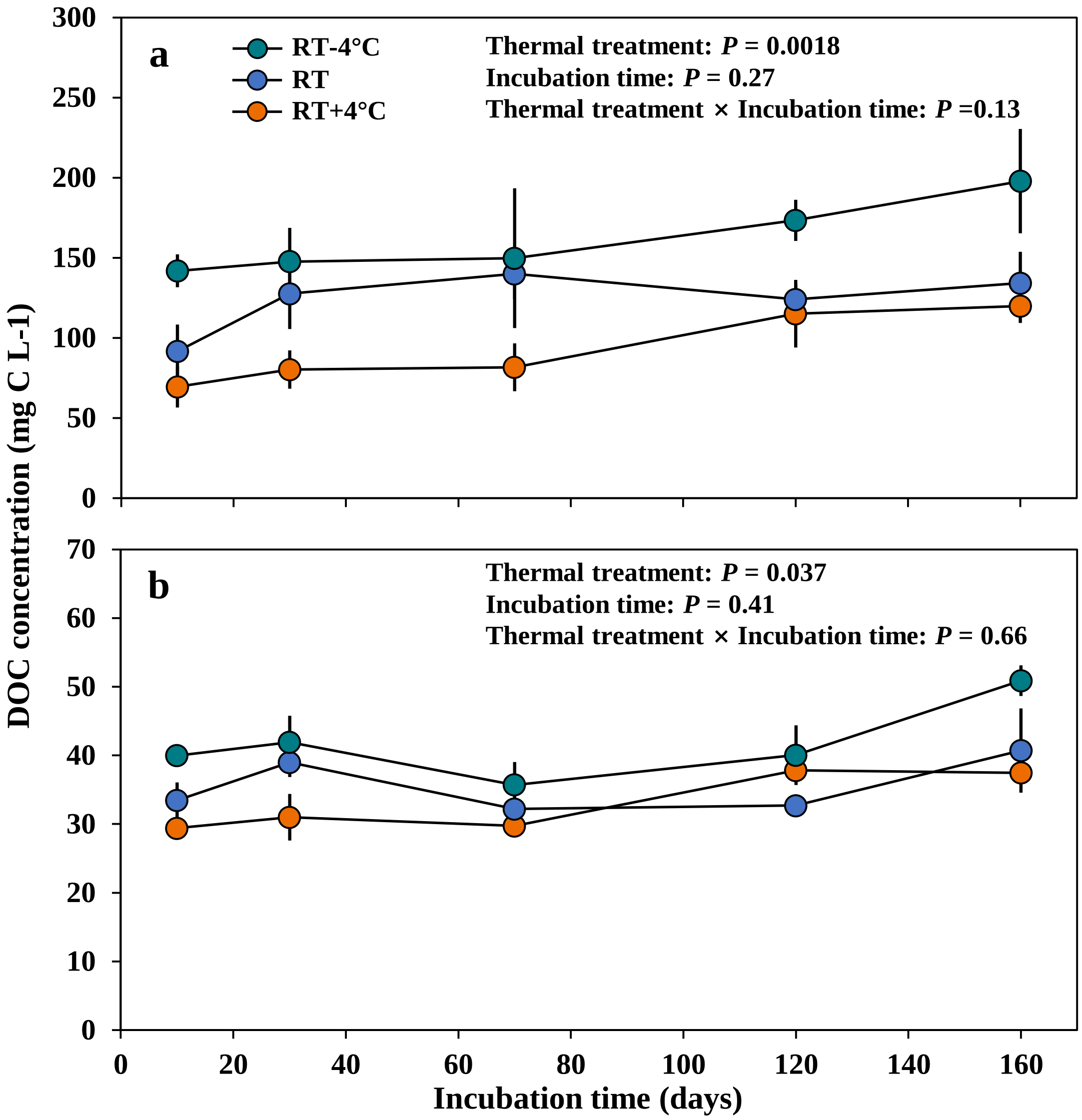

Supplementary Figure 5. Changes in dissolved organic carbon concentration during the main incubation period under the different thermal treatments. Using a general linear model, repeated-measures ANOVA was implemented to test for differences in DOC concentration attributable to incubation time, thermal treatment, and their interaction. Dissolved organic carbon (DOC) concentrations significantly increased (GKR: $P < 0.01$; TP: $P < 0.05$) with warming and were not affected by the incubation duration (both $P > 0.05$). **a**, the Great Khingan Range (GKR); **b**, the Tibetan Plateau (TP). RT, reference temperature (12°C). Data are presented as mean values ± SEM, n = 4 independent soil samples.

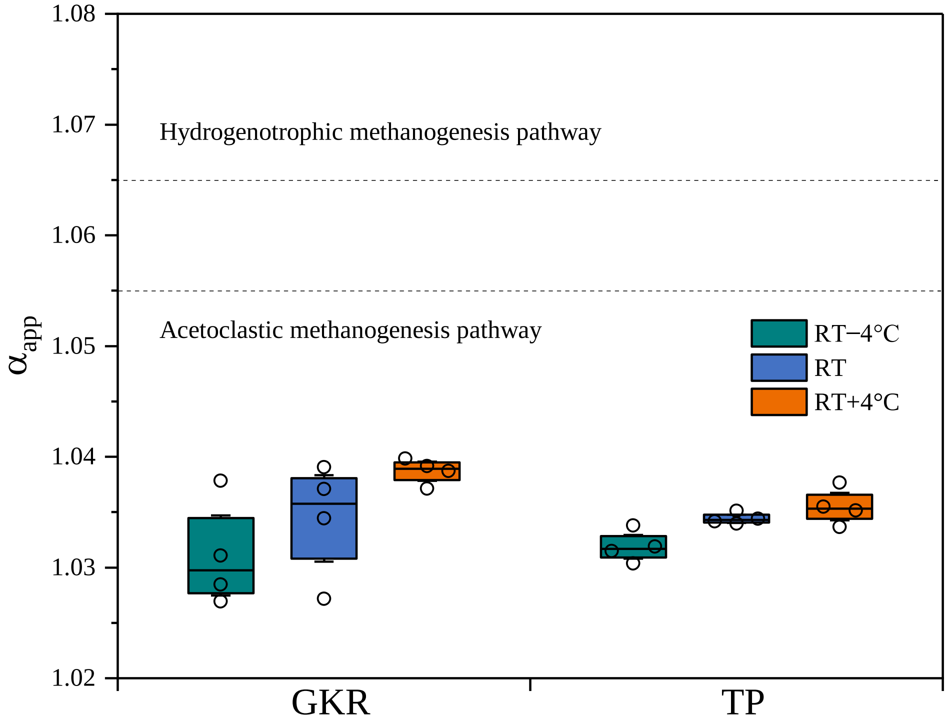

Supplementary Figure 6. Apparent carbon isotopic fractionation factor values for wetland soils. The apparent carbon isotopic fractionation factor (α_{app}) was calculated as ($\delta^{13}CO_2 + 10^3$)/($\delta^{13}CH_4 + 10^3$). It is generally accepted that $\alpha_{app} > 1.065$ and $\alpha_{app} < 1.055$ are characteristic of environments dominated by CO_2-dependent and acetate-dependent methanogenesis, respectively. The values of α_{app} measured in the incubation experiment ranged between 1.026 and 1.040, indicating that acetoclastic methanogenesis was the dominant methanogenic pathway in these wetland soils. GKR, the Great Khingan Range; TP, the Tibetan Plateau. Lines in boxes represent median. Bottom and top of boxes represent first and third quartiles, respectively. n = 4 independent soil samples.

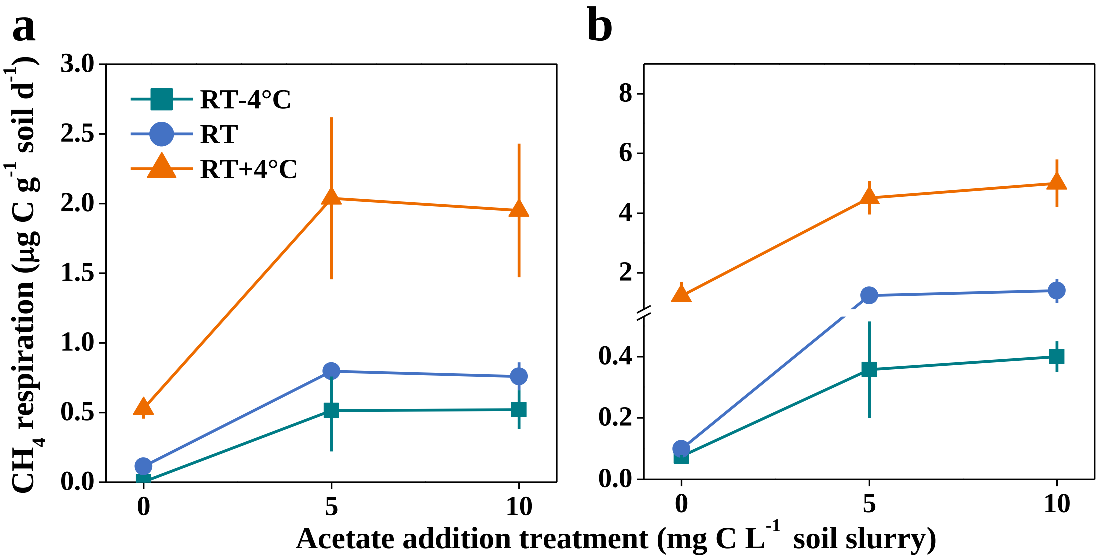

Supplementary Figure 7. Effect of acetate addition on soil CH_4 respiration rates. Acetate addition at a dose of 5 mg C L^{-1} soil slurry was sufficient for microbial CH_4 respiration over the short term (within 24 hours). **a**, the Great Khingan Range; **b**, the Tibetan Plateau. RT, reference temperature (12°C). Data are presented as mean values ± SEM, n = 4 independent soil samples.

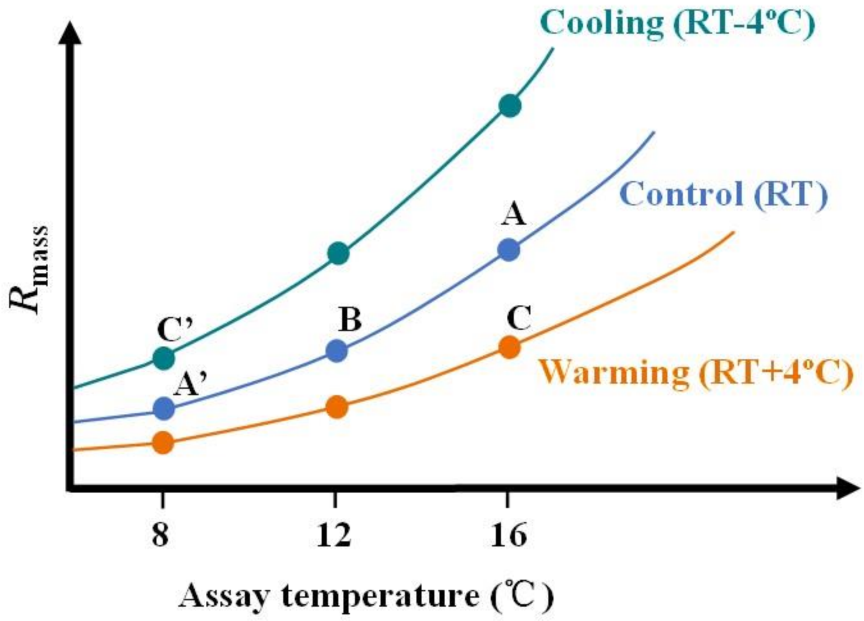

Supplementary Figure 8. Theoretical temperature response curves of soil mass-specific respiration under experimental warming and cooling. The reference temperature (RT) was set at 12°C (Fig. 1). Under experimental warming, the magnitude of compensatory responses (MCR) is a measure of how much of the respiratory increase (e.g., A minus B) expected due to short-term warming (minutes to hours) is eliminated by physiological compensation (e.g., A minus C) of R_{mass} under warming to the same extent but over a period of months. Thus, the MCR under experimental warming was calculated as follows: $MCR_{warming}$ = (A–C)/(A–B). As compensatory community responses, they must show opposite effects of warming and cooling, and the MCR under experimental cooling was thus calculated as follows: $MCR_{cooling}$ = (A′–C′)/(A′–B).

Supplementary Tables

Supplementary Table 1. Description of the study regions, the evaluated soil types and associated environmental parameters.

Soil information	GKR	TP
Region	Greater Khingan Range	Tibetan Plateau
Latitudinal range	52°25′N–53°21′N	37°06′N–37°42′N
Longitudinal range	122°01′E–124°20′E	101°05′E–101°46′E
Elevation range (m)	350–500	3100–3400
Total C (%)	43.75 ± 2.01	22.72 ± 1.60
Total N (%)	2.47 ± 0.23	1.6 ± 0.08
C:N ratio	18.16 ± 1.34	14.23 ± 0.79
Total S (%)	0.06 ± 0.02	0.37 ± 0.01
pH	5.5 ± 0.1	8.0 ± 0.1
Climate type	Cold temperate monsoon	Mountain plateau
MAT (°C)	−4.3	−2.0
MGT (°C)	13.2	11.5

MAT, mean annual temperature; MGT, mean growing-season temperature.

Supplementary Table 2. Comparison of the Q_{10} values of mass-specific CH_4 respiration (CH_4-R_{mass}) under experimental warming or cooling by 4°C. Values are the means ± SEM, n = 4 independent soil samples.

Wetland soils	Q_{10} of CH_4-R_{mass}			*P*
	RT−4°C	RT	RT+4°C	
GKR	1.37 ± 0.10	1.17 ± 0.04	1.24 ± 0.05	0.17
TP	1.83 ± 0.10	1.63 ± 0.17	1.48 ± 0.13	0.39

The Q_{10} values for each wetland type were treated statistically by one-way ANOVA, and pairwise multiple comparisons among the three thermal treatments were conducted using the Tukey HSD test at $P < 0.05$. RT, reference temperature (12°C). GKR, the Greater Khingan Range; TP, the Tibetan Plateau.
