# Supplementary material for: The thermal response of soil microbial methanogenesis decreases in magnitude with changing temperature
Source: Nat Commun. 2020 Nov 12;11:5733. doi: 10.1038/s41467-020-19549-4 (PMC7665204; doi:10.1038/s41467-020-19549-4)
Supplement: Supplementary file 3 — Reporting Summary [file 41467_2020_19549_MOESM3_ESM.pdf]

nature research

Corresponding author(s): Ming Nie

Last updated by author(s): Oct 13, 2020

Reporting Summary

Nature Research wishes to improve the reproducibility of the work that we publish. This form provides structure for consistency and transparency in reporting. For further information on Nature Research policies, see our Editorial Policies and the Editorial Policy Checklist.

Statistics

For all statistical analyses, confirm that the following items are present in the figure legend, table legend, main text, or Methods section.

n/a	Confirmed	
☐	☒	The exact sample size (*n*) for each experimental group/condition, given as a discrete number and unit of measurement
☐	☒	A statement on whether measurements were taken from distinct samples or whether the same sample was measured repeatedly
☐	☒	The statistical test(s) used AND whether they are one- or two-sided<br>*Only common tests should be described solely by name; describe more complex techniques in the Methods section.*
☐	☒	A description of all covariates tested
☐	☒	A description of any assumptions or corrections, such as tests of normality and adjustment for multiple comparisons
☐	☒	A full description of the statistical parameters including central tendency (e.g. means) or other basic estimates (e.g. regression coefficient) AND variation (e.g. standard deviation) or associated estimates of uncertainty (e.g. confidence intervals)
☐	☒	For null hypothesis testing, the test statistic (e.g. *F*, *t*, *r*) with confidence intervals, effect sizes, degrees of freedom and *P* value noted<br>*Give P values as exact values whenever suitable.*
☒	☐	For Bayesian analysis, information on the choice of priors and Markov chain Monte Carlo settings
☒	☐	For hierarchical and complex designs, identification of the appropriate level for tests and full reporting of outcomes
☐	☒	Estimates of effect sizes (e.g. Cohen's *d*, Pearson's *r*), indicating how they were calculated

Our web collection on statistics for biologists contains articles on many of the points above.

Software and code

Policy information about availability of computer code

Data collection	No software was used for data collection.
Data analysis	R version 3.4.3, with packages lme4 v1.1-16, MuMln v1.40.4 and lmerTest v3.0-1.<br>Raw sequencing reads were processed with VSEARCH v.1.9.658 and QIIME v.1.9.1 software pipelines.

For manuscripts utilizing custom algorithms or software that are central to the research but not yet described in published literature, software must be made available to editors and reviewers. We strongly encourage code deposition in a community repository (e.g. GitHub). See the Nature Research guidelines for submitting code & software for further information.

Data

Policy information about availability of data

All manuscripts must include a data availability statement. This statement should provide the following information, where applicable:

- Accession codes, unique identifiers, or web links for publicly available datasets
- A list of figures that have associated raw data
- A description of any restrictions on data availability

The data supporting the results in this paper have been deposited at DOI 10.5281/zenodo.4082274; Sequence data generated in the present study were deposited in NABI GenBank Short Read Archive (SRA) under accession number PRJNA668471 and the National Omics Data Encyclopedia (NODE, http://www.biosino.org/node) under accession number OEP000738 (Project ID).

Field-specific reporting

Please select the one below that is the best fit for your research. If you are not sure, read the appropriate sections before making your selection.

☐ Life sciences ☐ Behavioural & social sciences ☒ Ecological, evolutionary & environmental sciences

For a reference copy of the document with all sections, see nature.com/documents/nr-reporting-summary-flat.pdf

Ecological, evolutionary & environmental sciences study design

All studies must disclose on these points even when the disclosure is negative.

Study description	We conducted an anaerobic incubation study using wetland soils from the Greater Khingan Range and the Tibetan Plateau to investigate how a change in temperature affects the thermal response of microbial CH4 respiration through 160 days experimental warming and cooling, and to test whether these responses are related to a change in microbial community dynamics (See Fig. 1). At the start of the preincubation period, all microcosm bottles were randomly placed in a thermostatic bath at RT (± 0.1°C). On day 66, the bottles were randomly divided into three equivalent aliquots; two aliquots were transferred to separate thermostatic baths set at RT+4°C and RT−4°C, and the other aliquot was maintained in the thermostatic bath at RT (see Fig. 1b). A total of 336 bottles (2 wetlands × 4 sites × 3 thermal treatments × 14 soil replicates) were used for the 160-day main incubation period. The 14 replicates were randomly assigned to different analyses for the determination of the soil dissolved organic carbon (DOC) concentration (5/14), soil pH (2/14), microbial CH4 respiration (6/14), and microbial community (1/14).
Research sample	Soil samples were collected from the Greater Khingan Range (GKR) and the Tibetan Plateau (TP). Because of the differences in the soil methanogenic community and physicochemical properties between the selected GKR and TP soils (see Supplementary Table 1), there might be considerable differences in the thermal responses of methanogens to temperature change in these contrasting soils, and the use of these soils may convincingly test the compensatory response of microbial CH4 respiration to changing temperature and its underlying mechanisms.
Sampling strategy	Soil samples were collected from four sites (n = 4 independent soil samples) in the Greater Khingan Range and four sites on the Tibetan Plateau. We conducted soil sampling in June and July, 2016. At each site, we established a 20 m × 20 m square plot and collected 3 surface (0–20 cm) soil cores at each corner and in the center along a diagonal line. The soils from each site were homogenized by sieving to produce a composite sample. This sampling strategy allowed us to test the compensatory response of microbial CH4 respiration to changing temperature.
Data collection	Hongyang Chen and Ting Zhu conducted the overall experiment and measurements with the assistance from lab assistants (e.g., Chenhao Zhou and Jia Yao). Soil CH4 respiration measurements were obtained using cavity ring-down spectroscopy (Picarro G2201-i, USA). Methanogenic microbial biomass and community composition were determined using a common qPCR technique (LightCycler 96, Roche Molecular Systems, USA) and an Illumina MiSeq instrument (Illumina, USA).
Timing and spatial scale	Timing scale: Soils were incubated for 226 days (Preincubation period: 66 days, Main incubation period: 160 days) ranging from May 2017 to Jan 2018. All microcosms were subjected to 66 days of preincubation to allow the CH4 respiration rates to completely stabilize with the depletion of inorganic inorganic terminal electron acceptors (Supplementary Fig. 4). According to previous studies (see main text), the main incubation length under such conditions has been hypothesized to allow the compensatory thermal response of microbial respiration to occur.<br>Spatial scale: The wetlands of the Greater Khingan Range and Tibetan Plateau. There are marked differences in soil methanogenic community and physicochemical properties between these two wetland soils (see Supplementary Table 1).
Data exclusions	We did not exclude any data.
Reproducibility	In the whole experiment, all the findings can be replicated as all the incubation and measurement techniques are widely used and can be reproduced. For example, soil CH4 respiration measurements were obtained using cavity ring-down spectroscopy (Picarro G2201-i, USA) and methanogenic community composition were determined using a Illumina MiSeq technique. Information about the methods used in this paper are included in our material and methods.
Randomization	A total of 336 bottles (2 wetlands × 4 sites × 3 thermal treatments × 14 soil replicates) were used. The 14 replicates were randomly assigned to different analyses for the determination of the soil dissolved organic carbon (DOC) concentration (5/14), soil pH (2/14), microbial CH4 respiration (6/14), and microbial community (1/14).
Blinding	Our graduate research assistants had no idea what the bottle labels meant. In addition, microbial community composition was measured at a different institution where the investigators had only a sample number and hence no knowledge of where the sample came from.

Did the study involve field work? ☒ Yes ☐ No

Field work, collection and transport

Field conditions	Selected wetlands in the Greater Khingan Range --- mean growing-season temperature: 13.2°C; mean annual precipitation: 452 mm.<br>Selected wetlands on the Qinghai-Tibet Plateau --- mean growing-season temperature: 11.5°C; mean annual precipitation: 380 mm.<br>More details see Supplementary Table 1.

Location	Selected wetlands in the Greater Khingan Range --- Latitude: 52°25′N to 53°21′N, Longitude: 122°01′E to 124°20′E, elevation: 350 to 500 m.<br>Selected wetlands on the Qinghai-Tibet Plateau --- Latitude: 37°06′N to 37°42′N, Longitude: 101°05′E to 101°46′E, elevation: 3100 to 3400 m.
Access & import/export	No permit was required - all field work was performed around research stations. Soil samples were sealed in sterile polypropylene bags and transported to laboratory using iceboxes.
Disturbance	No disturbance.

Reporting for specific materials, systems and methods

We require information from authors about some types of materials, experimental systems and methods used in many studies. Here, indicate whether each material, system or method listed is relevant to your study. If you are not sure if a list item applies to your research, read the appropriate section before selecting a response.

Materials & experimental systems

n/a	Involved in the study	
☒	☐	Antibodies
☒	☐	Eukaryotic cell lines
☒	☐	Palaeontology and archaeology
☒	☐	Animals and other organisms
☒	☐	Human research participants
☒	☐	Clinical data
☒	☐	Dual use research of concern

Methods

n/a	Involved in the study	
☒	☐	ChIP-seq
☒	☐	Flow cytometry
☒	☐	MRI-based neuroimaging
